# The Lysosomotropic Activity of Hydrophobic Weak Base Drugs is Mediated via Their Intercalation into the Lysosomal Membrane

**DOI:** 10.3390/cells9051082

**Published:** 2020-04-27

**Authors:** Michal Stark, Tomás F. D. Silva, Guy Levin, Miguel Machuqueiro, Yehuda G. Assaraf

**Affiliations:** 1 The Fred Wyszkowski Cancer Research Laboratory, Department of Biology, Technion-Israel Institute of Technology, Haifa 3200003, Israel; bimichal@technion.ac.il (M.S.); guylevin@campus.technion.ac.il (G.L.); 2 BioISI-Biosystems & Integrative Sciences Institute, Faculdade de Ciências da Universidade de Lisboa, Campo Grande, 1749-016 Lisboa, Portugal; tfsilva@ciencias.ulisboa.pt (T.F.D.S.); machuque@ciencias.ulisboa.pt (M.M.)

**Keywords:** lysosomes, lysosomotropic drugs, drug sequestration, membrane intercalation, molecular dynamics

## Abstract

Lipophilic weak base therapeutic agents, termed lysosomotropic drugs (LDs), undergo marked sequestration and concentration within lysosomes, hence altering lysosomal functions. This lysosomal drug entrapment has been described as luminal drug compartmentalization. Consistent with our recent finding that LDs inflict a pH-dependent membrane fluidization, we herein demonstrate that LDs undergo intercalation and concentration within lysosomal membranes. The latter was revealed experimentally and computationally by (a) confocal microscopy of fluorescent compounds and drugs within lysosomal membranes, and (b) molecular dynamics modeling of the pH-dependent membrane insertion and accumulation of an assortment of LDs, including anticancer drugs. Based on the multiple functions of the lysosome as a central nutrient sensory hub and a degradation center, we discuss the molecular mechanisms underlying the alteration of morphology and impairment of lysosomal functions as consequences of LDs’ intercalation into lysosomes. Our findings bear important implications for drug design, drug induced lysosomal damage, diseases and pertaining therapeutics.

## 1. Introduction

Lysosomes are terminal degradation centers for various proteins and organelles in eukaryotic cells; however, they have emerged in recent years as central cellular sensory hubs regulating differentiation, cell division and apoptosis in response to multiple cues [1,2]. Lysosomes have been shown to promote tumorigenesis and cancer progression [3,4] and have been implicated in various anticancer drug resistance mechanisms [5]. Impaired lysosomal function can lead to various non-malignant lysosomal storage diseases (LSDs) [6]. Although LSDs are hereditary, lysosomal dysfunction can also arise following drug treatment, which may alter the morphology, size and pH of lysosomes [7,8,9]. The majority of lysosomes have an acidic luminal pH between 4.7 and 4.9 [10,11] and hence entrap a vast array of prognostic and therapeutic agents, all of which are hydrophobic weakly basic compounds [5] termed lysosomotropic drugs (LDs) [12]. Previous studies have found that LDs display a common denominator: beyond marked sequestration in lysosomes, LDs inflict lysosomal membrane permeabilization (LMP) [13,14,15,16,17]. In this respect, we, as well as others, have shown that multiple compounds that induce membrane permeabilization are typically *bona fide* membrane fluidizing agents which enhance the passive diffusion rates of lipid-soluble compounds and drugs [18,19,20,21]. Specifically, sunitinib (SUN) and siramesine (SIR), which we have shown to enhance membrane fluidity [22], are both known to induce LMP [23,24].

Recent studies have shown the marked sequestration of anticancer LDs in lysosomes, including nintedanib (NTD), a tyrosine kinase inhibitor (TKI) used against non-small cell lung cancer [25]; SUN, an antiangiogenic TKI used in the first line treatment of renal cell carcinoma [26,27]; daunorubicin (DNR), a DNA intercalator used in the treatment of acute myeloid leukemia [28]; and the MEK inhibitors trametinib and refametinib, used in the treatment of pancreatic ductal adenocarcinoma [29]. However, although the lysosomal compartmentalization of structurally and mechanistically distinct LDs is well documented, it is referred to as luminal drug sequestration [12]. Herein, we aimed at demonstrating that LDs accumulate in lysosomes via their intercalation into lysosomal membranes. Along this vein, original studies have demonstrated the concentration of ionizable hydrophobic drugs in cell membranes, liposomes and lipids [30,31,32]. The concentrations of LDs in both the lysosomal limiting membrane (LLM) and intra-lysosomal vesicles (ILVs) were revealed by confocal laser microscopy images of fluorescent dyes and chemotherapeutic drugs, as well as the constant-pH molecular dynamic (CpHMD) modeling of the pH-dependent membrane insertion and accumulation of an assortment of therapeutic LDs. These findings have important implications for drug-induced lysosomal alterations, lysosomal diseases, novel drug design and pertaining targeted therapeutics.

## 2. Materials and Methods

### 2.1. Chemicals

The central nervous system acting drugs (CNSDs) ethopropazine (Ethop, profenamine), clomipramine (Clomp, anafranil) and pimozide (Pimo), as well as DNR, chloroquine (CHQ) and the DNA dye Hoechst 33342, were from Sigma Aldrich (St. Louis, MO, USA). NTD was from Enzo life sciences (New York, NY, USA). The viable fluorescent lysosomal probes LysoTracker Red DND-99 (LTR) and LysoTracker Green DND-26 (LTG) were from Life Technologies (Grand Isle, NY, USA). Vacuolin-1 was from Santa Cruz Biotechnology (Dallas, TX, USA).

### 2.2. Tissue Culture

Human osteosarcoma U2OS cells (American Tissue Culture Collection, Manassas, VA, USA) were maintained in RPMI-1640 medium supplemented with 10% fetal bovine serum (Gibco, Life Technologies), 2 mM glutamine, 100 units/mL penicillin G and 100 μg/mL streptomycin sulfate (Biological Industries, Beit-Haemek, Israel) in a humidified atmosphere of 5% CO_2_ at 37 °C. U2OS cells stably expressing a GFP-tagged lysosomal associated membrane protein 1 (LAMP1-mGFP, a gift from Esteban Dell’Angelica, Addgene plasmid # 34831) [33] were grown in the presence of 650 µg/mL G418 (Calbiochem, EMD Chemicals, San Diego, CA, USA). The Clomp-resistant subline Clomp^R^ was selected and maintained under 10 µM Clomp.

### 2.3. Growth Inhibition Assay

U2OS cells were seeded in 96-well plates at 1000 cells/well in triplicate (in 0.1 mL of growth medium/well). The following day, CNSDs were added at 1/3 log increasing concentrations (0–100 µM), and cell survival was determined after 72 h, using a colorimetric cell proliferation kit according to the instructions of the manufacturer (XTT, Biological Industries). Percent cell survival was calculated relative to drug-free controls.

### 2.4. Live Imaging

#### 2.4.1. Confocal Live Imaging of Lysosomal Size and Number

Day 1: U2OS and Clomp^R^ cells were seeded in Eppendorf’s black Cell Imaging 24-well plates (Hamburg, Germany), with Clomp^R^ cells immediately supplemented with 10 µM Clomp. Day 2: CNSDs were added to U2OS cells at 10 µM (Clomp and Ethop) or 3 µM (Pimo), with 0.1% DMSO (the drug solvent) used for the drug-free control. Day 3: CHQ was added to U2OS cells at 20 µM. Day 5: Cells were washed with fresh medium and incubated for 45 min in the presence of 300 nM LTR and 1 µg/mL Hoechst 33342 in the dark. Cells were then washed with fresh growth medium and visualized using a confocal Zeiss LSM 710 inverted microscope (×63 magnification, Oberkochen, Germany) during incubation at 37 °C in an atmosphere of 5% CO_2_.

#### 2.4.2. InCell Lysosomal Quantification Analysis

**Figure 1:**
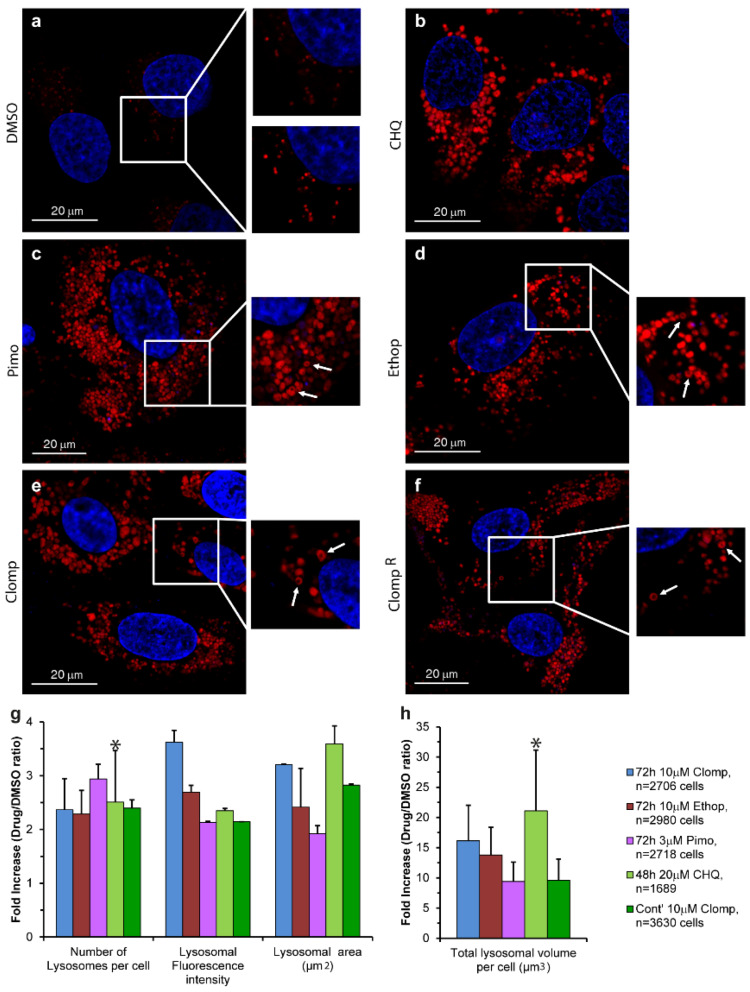
The effect of central nervous system acting drugs (CNSDs) on the number and size of lysosomes. U2OS cells were seeded in black glass bottom plates and treated for 72 h with either 10 µM Clomp, 10 µM Ethop, 30 µM Pimo or 0.1% DMSO for the drug free control. In addition, Clomp^R^ cells continuously grown in 10 µM Clomp were used, as well as U2OS cells treated for 48 h with 20 µM CHQ as a positive lysosomotropic control. Nuclei were stained with the viable DNA dye Hoechst 33342 (blue fluorescence), and lysosomes with the lysosomal probe LysoTracker Red DND-99 (LTR) (red fluorescence). (**a**–**f**): Representative images captured using a confocal Zeiss LSM 710 microscope (×63 magnification). Insets: (**a**) show the hardly visible drug free control lysosomes (upper inset) and over-enhanced lysosomes for better visualization (bottom inset), while (**b**–**f**) show examples of ring-like structures indicated by white arrows. All LTR images are representative of data collected from at least three independent experiments. (**g**,**h**) Cells were captured using an InCell Analyzer 2000 fluorescence microscope, and lysosomes were analyzed using the InCell Investigator software. Histograms depict the average median values obtained from three independent experiments ± S.D. All *p* values < 0.045 except for the ones indicated by an asterisk. See also Figure S3.

Cells were seeded in duplicate and treated as described above. Fluorescence was then visualized and recorded by an InCell Analyzer 2000 fluorescence microscope (version 3.7.3, GE Healthcare Bio-Sciences, Pittsburgh, PA, USA) during incubation at 37 °C in an atmosphere of 5% CO_2_. Sixteen random fields were captured from each well (i.e., 32 fields per treatment). Several parameters, including the number of lysosomes per cell, their fluorescence intensity and lysosome area were determined using the InCell Investigator software. Results were obtained as mean numbers per field. Total cellular lysosomal volume was calculated as follows: lysosome radii (r) were mathematically derived from the mean lysosomal area (A), A = πr^2^. Then, lysosomal volume (V) was calculated as V = 4/3 πr^3^. Finally, the volume values were multiplied by the number of lysosomes per cell. The experiment was performed three independent times. Figure S3 depicts the results of a representative experiment, while Figure 1g–h depicts the mean values of all three experiments ± SD.

#### 2.4.3. Confocal Visualization of Fluorescent Compounds in Lysosomal Membranes

For the enlargement of lysosomes, we utilized either vacuolin-1 or Clomp. Parental U2OS and Clomp^R^ cells were seeded in Eppendorf’s black Cell Imaging 24-well plates. The following day, prior to confocal imaging, the growth medium was replaced and the cells were treated with 1 µM vacuolin-1 for 2 h and supplemented with either 300 nM LTR, 300 nM LTG, 10 µM DNR or 10 µM NTD for the last 45 min. Cells were washed with fresh growth medium and visualized using a confocal Zeiss LSM 710 inverted microscope (×63 magnification) during incubation at 37 °C in an atmosphere of 5% CO_2_.

LAMP1-mGFP, LTG and NTD were excited at 488 nm, LTR and DNR at 514 nm and Hoechst 33342 at 405 nm. Confocal microscopy images were processed using the ZEN (2.3 black edition) software. Fluorescence measurements along the diameters of individual lysosomes were performed using the ImageJ software (version 1.52i, Wayne Rasband National Institute of Health, Washington, DC, USA).

### 2.5. CpHMD Simulations

#### 2.5.1. System Setup and Simulation Settings

We adapted the lipid bilayer composed of 128 molecules of 1,2-dimyristoyl-sn-glycero-3-phosphocholine (DMPC) lipids from a previous study [34] and built a new system for each drug, i.e., Pimo, Ethop, Clomp, DNR, NTD, SUN and vincristine (VCR). The molecules were placed in bulk water at approximately 15 Å away from the membrane surface, to allow for unbiased membrane insertion in every replicate. LDs are apolar compounds that, when placed in a vicinity of a membrane, tend to interact avidly with lipids and insert into the lipid bilayer in an attempt to minimize the contact between their hydrophobic parts and water. For simplicity and due to membrane symmetry, we can distinguish three preferred positions for the LDs: (1) being away from the membrane, at the water phase, (2) residing at the water/membrane interface, stabilized by some electrostatic interactions with the lipid head groups, and (3) inserted deeply inside the membrane, hence minimizing the contact with water. In our simulations, the first position is rather undesirable (most molecules are significantly apolar) and, after some equilibration time, the compounds are already exchanging between positions 2 and 3. VCR and DNR are more polar and, hence, require significantly longer times for the simulations to equilibrate and allow for membrane insertion events. To obtain meaningful data for insertion statistics, we extended the normal initial equilibration time for the compounds (i.e., 100 ns), twice for VCR and three times for DNR.

Molecular dynamics (MD) simulations were carried out using the GROMACS 4.0.7 package [35]. The GROMOS 54A7 force field [36] was used together with the SPC water model [37]. The initial topologies for all drugs were built with the Automated Topology Builder (ATB) [38] and manually curated. These topologies were modified in the pairs section in order to exclude all 1–4 interactions in the aromatic moieties, analogously to the GROMOS rules for conjugated rings. The charge sets were derived from Merz-Singh-Kollman analysis using the electrostatic potential calculated in structures optimized with Gaussian 09 [39] using the B3LYP functional [40] and 6–31G* basis set. The compounds were analyzed in both neutral and protonated (cationic) states.

#### 2.5.2. Poisson-Boltzmann/Monte Carlo Calculations

The Poisson-Boltzmann (PB) calculations were performed using Delphi V5.1 [41], using the radii calculated from the GROMOS 54A7 Lennard-Jones parameters [42]. The molecular surface of the system (DMPC + drug) was defined using a probe with a radius of 1.4 Å, while the ion exclusion layer was of 2.0 Å and the ionic strength was set to 0.1 M. The dielectric constants used were 2 and 80 for the solute and solvent, respectively. Each PB calculation requires a two-step focusing procedure where, initially, we centered the titrating group in a cubic grid with 61 points with approximately 1 Å spacing (coarse grid), followed by a focusing step, reducing the spacing between grid points to 0.25 Å (focus grid). The relaxation factors used in the linear and nonlinear iteration processes for the coarse grid were 0.20 and 0.75, respectively. Periodic boundary conditions were set for the x and y directions. Background interactions were calculated up to a 25 Å cut-off, with a convergence threshold of 0.01 for the electrostatic potential.

Monte Carlo calculations were performed with the PETIT program (version 1.6) [43] using the free-energy terms obtained from the PB calculations and to sample the protonation states of each compound.

#### 2.5.3. pH Replica Exchange Settings

The pH replica exchange (pHRE) method [44] is based on the stochastic titration constant-pH MD method (CpHMD) [45], which enables the treatment of pH effects as an external parameter in the MD simulation, while allowing frequent exchange attempts of pH values between two adjacent replicas (CpHMD simulations). Five replicates of 200 ns simulations were performed for all systems using pHRE. The simulations were extended to 300 and 500 ns for VCR and DNR, respectively, in order to improve their sampling. Each replicate is composed of four replicas, which were assigned an initial pH value from a molecule specific pH range: Clomp and Ethop (7.0 to 10.0), Pimo (6.3 to 9.3), SUN and NTD (5.5 to 8.5), VCR (5.7 to 8.7) and DNR (7.6 to 10.6), with a 1.0 pH unit step. The frequency of exchange attempts was every 20 ps.

#### 2.5.4. Membrane Insertion Procedure

To perform p*K*_a_ calculations along the membrane normal for each LD, we separated our conformations according to the relative insertion of their titrable amino groups against the average Z position of the membrane’s phosphate group atoms that are within a 6 Å radius from the compound in a two-dimensional plane (*x*/*y* axis). If, within this radius, a minimum of 10 phosphate group atoms (phosphorous and oxygen) is not obtained, then the absolute closest 10 atoms are used. This method accounts for possible local membrane deformations, since it uses the neighboring lipids to obtain these relative positions. After obtaining the insertion values for the titrating group in all conformations (please note that the insertion is not for the whole LD, but only for the titrating group), we separated each, and the respective protonation states, to an insertion bin. In each bin, we applied two criteria to assess sample quality before performing a fit to the Henderson-Hasselbalch (HH) equation. In the current study, each bin required a set of at least two different pH values from two different replicates to obtain their average protonation values. To obtain successful HH fits and good p*K*_a_ estimations, the data also need to display monotonicity (protonation must decrease with an increase in pH). These criteria allow for robust calculations of the p*K*_a_ values and of the standard error of the mean. All analyses were performed using in-house software and scripting (http://mms.rd.ciencias.ulisboa.pt/#software).

### 2.6. Statistical Analyses

For the quantification of changes in lysosomal parameters following CNSD treatment, a two-tailed two-sample unequal variance Student *t*-test was performed per experiment, and a two-tailed paired *t*-test was used for the average of the three experimental repeats. *p* values ≤ 0.05 were considered statistically significant and are mentioned in the figure legends.

The p*K*_a_ error values shown were obtained using a bootstrap approach [46]. However, to avoid fitting issues, a Bayesian bootstrap [47] was used where we ran 1000 bootstraps from our average protonation samples and, in each bootstrap, random weights for each sample were generated. For consistency, we also applied the two previous criteria to this procedure before calculating the final p*K*_a_ and error values. The protonation error values were calculated using a simple standard error of the mean.

## 3. Results

### 3.1. Live Imaging

We have recently proposed that hydrophobic, weakly basic LDs intercalate into the LLM via their lipophilic polyaromatic ring structure (illustrated in Figure S1), with their basic residues protruding into the acidic lysosomal lumen, where they undergo protonation [22]. Along this vein, using fluorescence recovery after photobleaching (FRAP), we recently demonstrated that markedly sequestered LDs inflict lysosomal membrane fluidization [22]. Towards deciphering the molecular mechanism underlying lysosomal membrane fluidization, we herein aimed at demonstrating the physical intercalation of weakly basic hydrophobic anticancer drugs and other therapeutic agents into the LLM. In order to enhance our ability to observe the lysosomal membrane confinement of LDs, we first expanded the lysosomal compartment using pharmacological agents without triggering cell death; for this purpose, we chose therapeutic LDs that are widely used as chronic treatments while displaying low toxicity. In this respect, most CNSDs are hydrophobic weak bases since they were strategically designed to cross the blood–brain barrier [48] and induce a long-lasting effect [49]. The CNSDs that we used here were as follows: Pimo, an anti-psychotic drug of the diphenylbutylpiperidine class [50], which was shown to induce the formation of autophagosomes and autolysosomes [51,52]; the tricyclic anti-depressant Clomp [53], which was identified as a lysosomotropic agent via a High Content Screening Assay for Identifying Lysosomotropic Compounds [54]; and the anti-Parkinsonian agent Ethop of the phenothiazine family [55], which, together with Pimo and Clomp, was identified as a functional inhibitor of acid sphingomyelinase, termed FIASMA [56,57], and was hence suggested to interact with the lysosomal membrane.

A 72 h cytotoxicity assay was performed on human osteosarcoma U2OS cells to assess the desired non-cytotoxic drug concentration for each CNSD, following which the IC_10_ (drug concentration resulting in a 10% growth inhibition) values were determined for Pimo, Ethop and Clomp (i.e., 3 µM, 10 µM and 10 µM, respectively). Using fluorescence microscopy imaging, we evaluated the lysosomotropic effects of the CNSDs on the number and size of lysosomes in U2OS cells treated for 72 h with the abovementioned concentrations of LDs. Cells were then viably stained with the established lysosomal marker LTR and analyzed using live confocal microscopy (Figure 1a–f). CHQ was used as a *bona fide* positive control for its well-established lysosomotropic activity that induces a marked expansion of the lysosomal compartment (Figure 1b) [58,59]. All three CNSDs induced a substantial increase in both lysosome size and lysosome number per cell, as reflected by the viably LTR-stained organelles (Figure 1, compare the red LTR fluorescence in b–f to in a). This was also corroborated with U2OS cells overexpressing a LAMP1-mGFP, yielding the same LLM staining of the enlarged lysosomes (Figure S2). One can notice the fluorescent ring-like structures that are captured by the laser scanning through the mid-section of lysosomes containing the green fluorescent LAMP1; the same fluorescent circular structures were observed with the viable LTR staining (Figure 1a–f, insets, white arrows), revealing the confinement of this lysosomal probe to the LLM, rather than to the lumen. These findings are in accord with our recent hypothesis that LDs accumulate in the LLM and could explain the LD-dependent membrane fluidization effect we recently uncovered [22]. Since these fluorescent circular organelle structures are difficult to visualize in the relatively small lysosomes under physiological conditions, we performed a quantitative analysis to evaluate the lysosomotropic effect of the CNSDs and determine which compound is the most effective in increasing lysosome size. We repeated the 72 h treatment with the CNSDs and undertook LTR staining and imaging, using an InCell fluorescence microscope and analyzer. The software can quantify the number of lysosomes per cell, their fluorescence intensity and their size (i.e., area in µm^2^). Based on these quantitative parameters, we calculated the total cellular lysosomal volume, as detailed under Materials and Methods (Figure 1g–h and Figure S3). While all three CNSDs induced remarkable effects, resulting in a median increase of 10–20-fold in the total lysosomal volume per cell, Clomp exhibited the strongest and most stable effect on lysosomal size (i.e., 3.2 ± 0.013-fold over the drug-free DMSO control, with a median area of 3.03 µm^2^, Figure S3). We therefore established a Clomp-resistant subline named Clomp^R^ by single step continuous selection with 10 µM Clomp. This subline displayed a stable expansion of the lysosomal compartment as long as cells were grown under drug selective conditions, although to a lesser extent than cells treated with Clomp for 72 h (Figure 1f–h).

**Figure 2:**
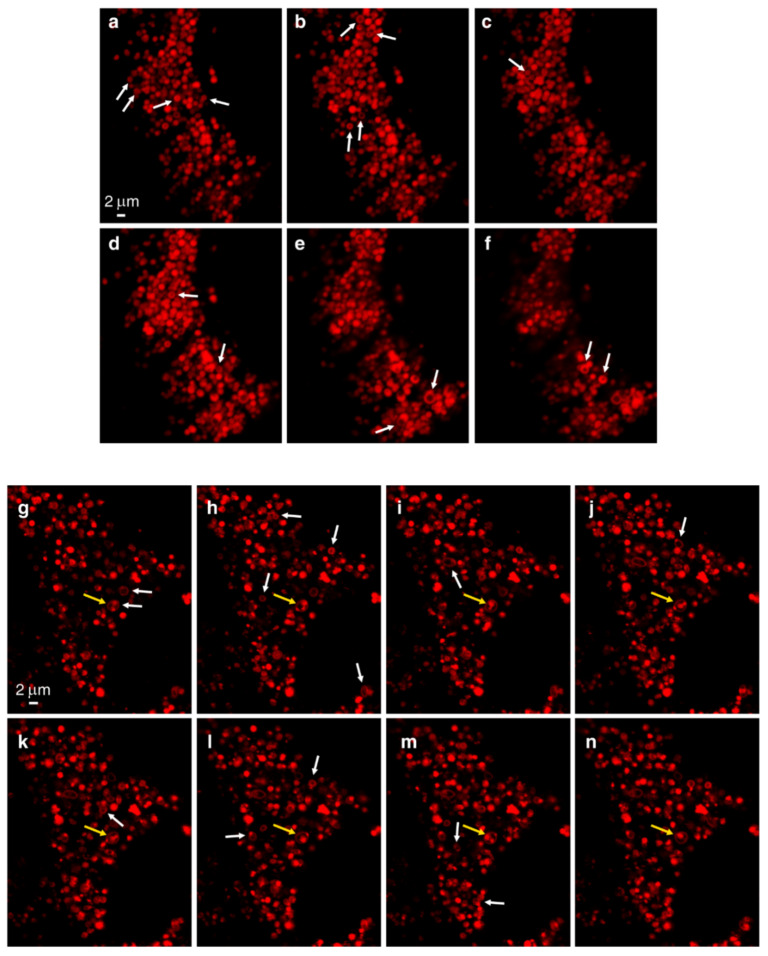
Z-Stack analysis of LTR-loaded lysosomes. Clomp^R^ cells (**a**–**f**) or vacuolin-1-treated U2OS cells (**g**–**n**) were labeled with LTR and scanned with a confocal Zeiss LSM 710 microscope (×63 magnification) using focus stacking with 0.2 µm slices. White arrows indicate ring-like structures as they first appear. The yellow arrow points to a lysosome harboring internal vesicles.

**Figure 3:**
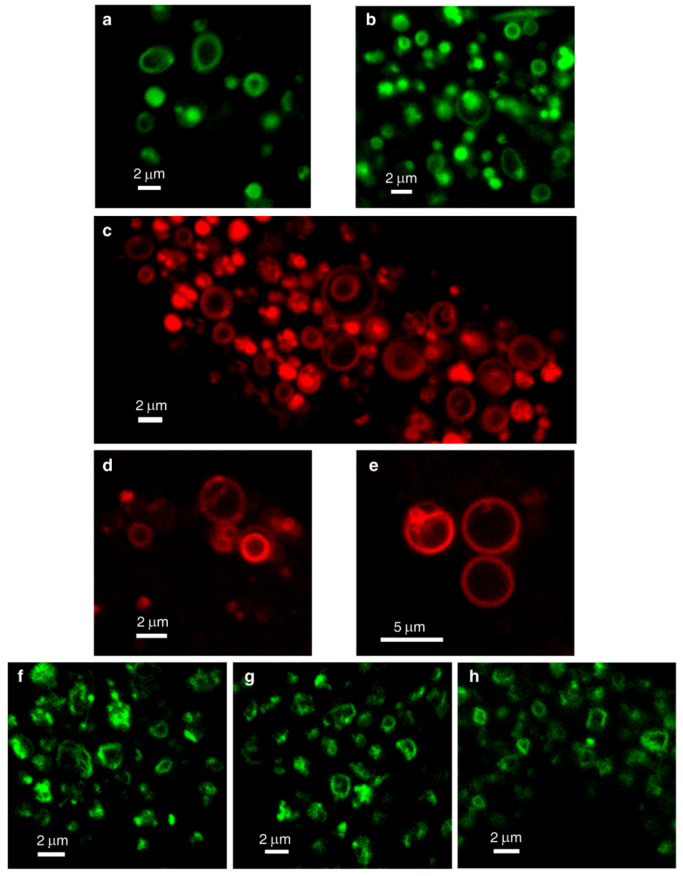
Lysosomal membrane staining with naturally fluorescent lysosomotropic compounds. Vacuolin-1-treated U2OS cells were loaded for 45 min with LysoTracker Green DND-26 (LTG) (**a**,**b**), daunorubicin (DNR) (**c**–**e**) or nintedanib (NTD) (**f**–**h**) and imaged with a confocal Zeiss LSM 710 microscope (×63 magnification). All images are representative of data collected from at least three independent experiments. See also Figure S4.

**Figure 4:**
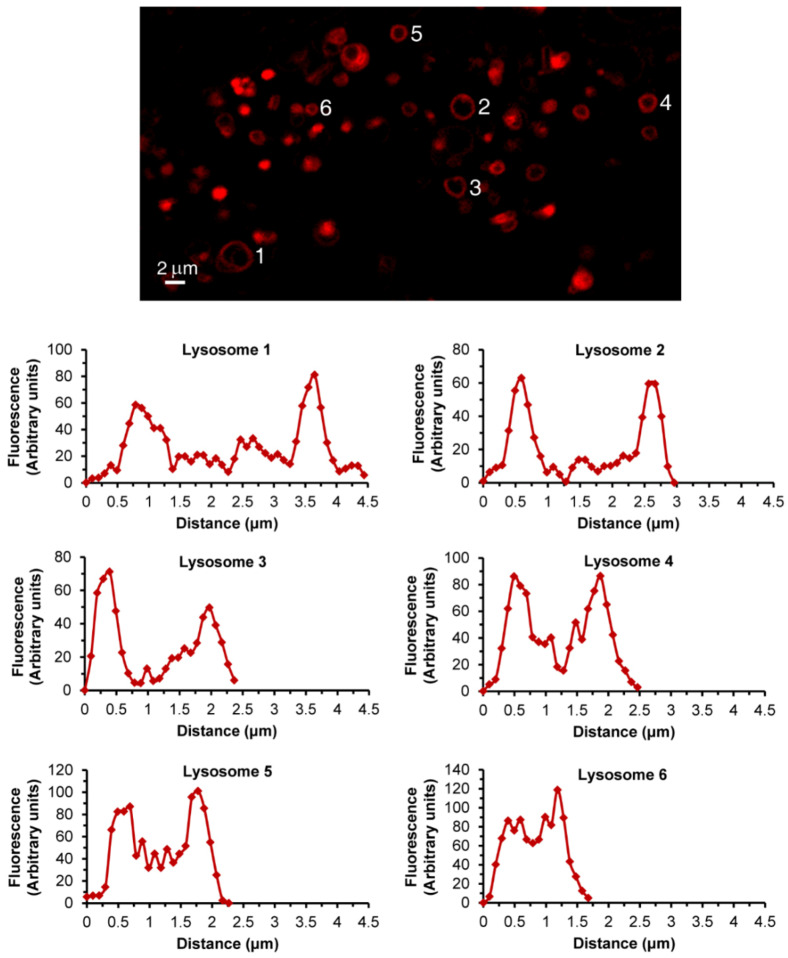
Lysosomal DNR fluorescence analysis. Vacuolin-1-treated U2OS cells were loaded for 45 min with DNR and imaged with a confocal Zeiss LSM 710 microscope (×63 magnification). Individual lysosomes were analyzed for fluorescence intensity along their diameter using the ImageJ software. Each graph corresponds to a lysosome numbered in the photo.

In order to demonstrate the LLM localization of *bona fide* fluorescent dyes and naturally fluorescent chemotherapeutic drugs, we utilized, in addition to Clomp, vacuolin-1. The latter is a small molecule that was shown to expand lysosomes by inhibiting the fusion between autophagosomes and lysosomes [60] and by inhibiting Ca^2+^-dependent lysosomal exocytosis [61]. Clomp^R^ cells were loaded with LTR and visualized by confocal microscopy using focus stacking to record lysosomal images at different focal planes (i.e., Z-stacks, Figure 2). As the scan proceeded through the lysosome-containing intracellular zone, the appearance of lysosomes shifted from a fluorescent ball-shape to a ring-like structure and back again (Figure 2a–f). The same visuals were captured in U2OS cells treated with vacuolin-1 (Figure 2g–n). However, vacuolin-1 appeared to produce larger lysosomes with thinner membranes (Figure 2, compare a–f to g–n). To ascertain that this finding was not a peculiar trait of the viable LTR dye, and to confirm the relevance of this finding to the clinic, we stained cells with expanded lysosomes with the fluorescent agent LTG (Figure 3a–b) and the naturally fluorescent FDA-approved chemotherapeutic LDs DNR (Figure 3c–e), a DNA intercalating agent [62], and NTD (Figure 3f–h), an oral tyrosine kinase inhibitor [63,64]. Both anticancer drugs displayed a marked incorporation into the LLM, as evident from the fluorescent ring-like structures. Expectedly, fluorescence microscopy analysis of individual lysosomes showed that the periphery of lysosomes i.e., the membrane, was brightly fluorescent with DNR. By contrast, a marked decrease in fluorescence within the lumen of lysosomes was noticeable, down to background levels, albeit the luminal fluorescence was sometimes above the background levels, especially when lysosomes were small. This fluorescence distinction between the brightly fluorescent LLM and the less fluorescent lysosomal lumen was much more prominent, the bigger the lysosomes were (Figure 4). However, unlike the symmetric round rings observed following LTR, LTG and DNR staining, NTD-loaded lysosomes displayed highly distorted non-circular morphologies with sharp edges and an unequal fluorescence distribution in the LLM (Figure 3, compare a–e to f–h). An additional interesting observation is the expansion of ILVs by vacuolin-1 (Figure 3c) and the apparent accumulation of DNR therein, also notable with LTR in Figure 2g–n (yellow arrow) and further examples in Figure S4.

### 3.2. CpHMD Simulations of LD Insertion into a Phospholipid Bilayer

**Figure 5:**
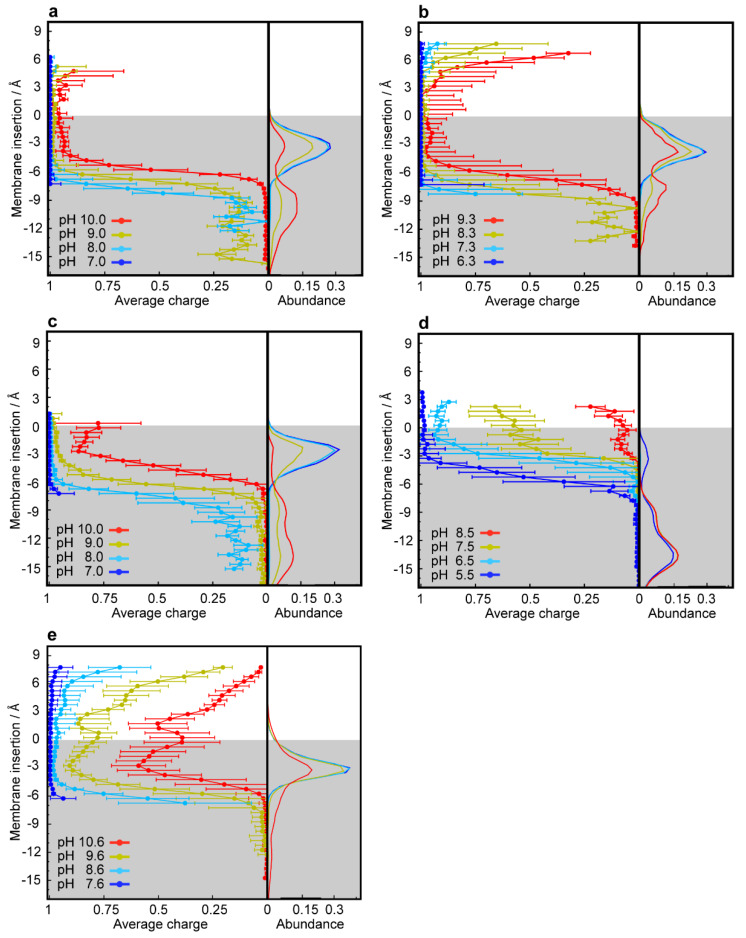
CpHMD simulations of the membrane insertion of lysosomotropic compounds. Average protonation (left subplots) and abundance histograms (right subplots) are depicted along the membrane insertion axis for Ethop (**a**), Pimo (**b**), Clomp (**c**), NTD (**d**) and DNR (**e**). The gray-shaded areas represent the membrane internal regions below the phosphate groups, which are used as insertion references (see Materials and Methods).

**Figure 6:**
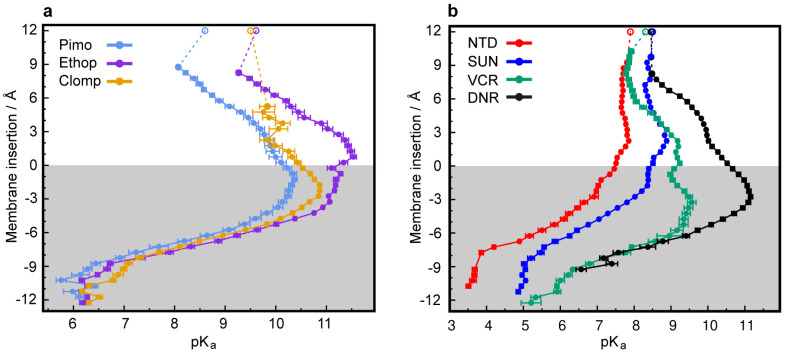
Predicted shifts in p*K*_a_ values during membrane insertion. p*K*_a_ profiles along the membrane normal for the CNSDs (**a**) and anticancer drugs (**b**). The unfilled points represent the experimental solution p*K*_a_ values (Figure S1). The gray-shaded areas represent the membrane internal regions below the phosphate atoms, which are used as insertion references (see Materials and Methods).

LDs are very hydrophobic weak bases, poorly soluble in water, with the ability to deprotonate and passively cross biological membranes. The pH values encountered in the different cellular compartments should have a major impact on the diffusion rates of these compounds across membranes. To address this point, we used CpHMD simulations coupled with a replica exchange scheme [44] of several LDs interacting with a membrane model at different pH values. The pH range used in each simulation was chosen to capture the p*K*_a_ shifts induced by the membrane environment according to the p*K*_a_ values of the LDs in water. The LDs were set up on the water phase to avoid any conformational bias towards the membrane. In our timescale, it was expected that these hydrophobic compounds would equilibrate at the water/membrane interface, which indeed occurred relatively quickly with most LDs. However, convergence could only be obtained with DNR after extending the duration of the simulations to 500 ns, which allowed membrane insertion events. DNR is more polar than the other LDs used in this study, and the energy barrier for its membrane insertion is higher, creating a kinetic trap, due to our initial unbiased set up protocol. In our simulations, no exchange of LDs between monolayers was observed, which is not surprising, since this is probably the rate-limiting step for the diffusion-based membrane crossing, occurring on a slower timescale. Consequently, the LD/membrane monolayer interactions model the drug accumulation in each type of membrane, dictated by the pH value at the water phase. We observed that deprotonation is a prerequisite for all compounds to allow for their membrane insertion, as evident from the decrease in the average charge of the LDs at deeper insertion depths (Figure 5a–e and Figure S5a–b, left subplots) and the shifts towards lower values in the p*K*_a_ profiles along the membrane insertion (Figure 6). A simple way of tracking the preferred positions of the LDs in the membrane is to analyze their insertion distributions, which we used to calculate the protonation and p*K*_a_ profiles (Figure 5a–e and Figure S5a–b, right subplots). It should be noted that these insertion values correspond only to the relative position of the titrable amino group and not the entire molecule, which, in this case, seems appropriate, since this is the only group that can “sense” pH. The membrane insertion graphs obtained reveal that pH and, consequently, the protonation state, have a major effect on the preferred membrane depth locations of the LDs. In their protonated forms, the CNSDs preferably accumulate slightly below the phosphate region of the phospholipids and, upon deprotonation, they undergo deeper insertion into the lipid bilayer, ~10 Å below the phosphate region (Figure 5a–c). Clomp and Ethop, at pH 7, and Pimo, at pH 6, became entrapped at the water/membrane interface (~3 Å below the phosphate atoms). The antitumor drugs SUN, VCR and DNR displayed similar insertion distributions and appear to be fully entrapped at the interface at pH 6.5, 6.7 and 9.6, respectively (Figure S5a–b and Figure 5e), indicating that below these pH values, insertion is highly unfavorable. NTD appears to deprotonate easily and inserts deeply into the membrane, and only at pH 5.5 (the lowest value we used for this LD) the protonated species was found to partially localize at the water/membrane interface (Figure 5d). We calculated the proton binding affinities along the membrane normal, which we termed p*K*_a_ values for simplicity, from the protonation data (Figure 6). These profiles illustrate how the p*K*_a_ values of these LDs markedly change upon interaction with the lipid bilayer, achieving shifts of 3–4 units below the water p*K*_a_ values. We selected several representative conformational snapshots of LDs interacting with the membrane to illustrate the preferred membrane locations (Figures S5c–d and S6). The selection process for representative LD conformational snapshots was based on the histogram peaks displayed in Figure 5 and Figure S5a–b which, consequently, correspond to the different energy minima. For each LD, the left panel shows a more inserted conformation typical of higher pH values, where the Lewis base is deprotonated, and the right panel shows the water/interface position, more common at the lower pH values, where most molecules are protonated and unable to penetrate the membrane.

## 4. Discussion

It is well documented that LDs readily traverse biomembranes and accumulate within lysosomes, hence altering lysosome size, morphology and function [65,66,67]. Specifically, LDs were reported to alter lysosomal enzyme levels and impair their function—however, the exact intra-lysosomal localization of LDs was not reported. To this end, we here demonstrate that LDs intercalate into lysosomal membranes and highly concentrate therein. We thus discuss how this membrane confinement of LDs underlies their lysosomotropic mechanisms of action.

The p*K*_a_ values of the hydrophobic weakly basic LDs studied herein are near or well above the physiological pH of ~7.4 (Figure S1, [64,68,69,70,71,72,73,74,75]), resulting in their partial, to almost total, protonation in the aqueous phase (i.e., in the bloodstream and cytosol). Protonation increases as they approach the membrane (i.e., lipid bilayer or lipid droplet) due to the stabilization of their charged form, as reflected by the upsurge in their p*K*_a_ values and average charge (Figure 5, Figure S5) [34,76]. Although the compounds have high log *p* values (Figure S1, [64,72,75,77,78,79]) and poor water solubility at this pH, a problem that is overcome in the bloodstream by their binding to the highly abundant serum albumin [80], the charged forms are unable to passively cross biological membranes. According to the pH-partition hypothesis, an ionizable molecule can only diffuse across lipid bilayer membranes in its neutral form [81]; hence, deprotonation is required during membrane insertion. Indeed, a drastic decline in the p*K*_a_ profiles of the LDs was observed, accompanied by the complete neutralization of their charges. Deprotonation is supported by the desolvation process occurring during membrane insertion as the contact with the aqueous phase diminishes, a process that stabilizes their neutral forms [34]. This effect has been shown for titrable amino acids during their insertion into a lipid bilayer, using a recently developed computational methodology, where the authors observed either a p*K*_a_ increase or decrease for the anionic or cationic groups, respectively [34].

Abundance data from the insertion analyses reveal that our tested LDs were predominantly confined to the membrane, and this was recently shown by molecular dynamics (MD) simulations and the COSMOmic approach for various amphiphilic molecules [82]. The authors’ calculations localized most compounds just below the phospholipid head groups, in agreement with our simulations herein. The LDs CHQ, imatinib and nilotinib were shown to accumulate in lysosomes at concentrations 1000-fold higher than their extracellular drug concentrations within 2 h [82]. Such high concentrations of positively charged drugs at the water-interface of the bilayer can alter the delicate electrostatic balance in the lipid headgroups, inducing repulsion between the choline groups of phospholipids and increasing the distance between neighbor lipids (area per lipid), which results in a marked increase in membrane fluidity [83]. The latter is in complete concordance with reports of increased membrane fluidity induced by SUN, SIR [22] and DNR [84], as well as with the observed LMP induced by LDs [85]. In both cases, the fluidization effect was pH-dependent, a finding that correlates with the current results, where the abundance of LDs at the water/membrane interface was more prominent at acidic pH, where all compounds have the highest charge due to immediate protonation. The latter requires that LDs reside, at least transiently, within the lipid bilayer, with their basic residues protruding into the acidic lysosomal lumen—hence, being in proximity to the aqueous phase is a prerequisite for protonation. Upon protonation, the weakly basic compounds are entrapped, since the energetic barrier for their diffusion across the bilayer center is too high. For example, the LD propranolol, in its protonated form, was calculated to face an energetic barrier of ~12 kcal/mol when encountering the hydrocarbon core of the bilayer [76]. Indeed, neutron diffraction experiments positioned propranolol within a DMPC lipid bilayer, with its naphthalene moiety partitioned into the hydrophobic core and its charged amine side chain in the phospholipid head group region [85]. The protonated charged form is further stabilized by forming a surface ion pair with the zwitterionic lipid headgroups [32,86], thus anchoring the molecule in place. Furthermore, pH-dependent ionic interactions between the weak base local anesthetic tetracaine and the head groups of phosphatidylcholine were demonstrated by conformational changes in the lipids’ head groups. This was inferred from changes in the NMR spectra of ^2^H in the trimethylammonium group and ^31^P in the phosphate group of phosphatidylcholine [87]. Indeed, CpHMD simulations at the lowest pH reveal that, except for NTD, our tested LDs do not penetrate the bilayer deep enough to cross over to the other side. In concordance with these experimental and computational results, we here show by confocal microscopy imaging the intra-membrane fluorescence of the lysosomotropic compounds LTR, LTG, DNR and NTD.

Fluorescent ring-like structures captured by confocal microscopy, following lysosomal expansion by Clomp or vacuolin-1, were easily detectable for all naturally fluorescent compounds. It should be noted that we did not use the naturally green fluorescent SUN for this fluorescence microscopy since it induces lysosomal photodestruction via the rapid formation of reactive oxygen species and lysosomal membrane rupture [27]. Beyond the need to use a laser scanning microscope, in order to capture the luminal regions of the lysosomes, the limiting factors in visualizing fluorescent lysosomal “rings” are lysosomal diameter, lysosomal membrane thickness and fluorescence intensity. The three parameters influence the actual ability to detect the reduction in fluorescence in the lumen area and distinguish between the lumen and the membrane region. In this respect, vacuolin-1 produced larger lysosomes with thinner membranes and lower fluorescence intensities than Clomp; hence, it is an optimal agent for the visualization of fluorescent lysosomal rings. The confined localization detected with the fluorescent molecules could only be achieved if they were to partition predominantly into the membrane compartment. As drug molecules accumulate at the water/membrane interface, they can detach completely from the bilayer and move into the lysosomal lumen. This can be interpreted from Figure 3c and Figure S4, presenting intra-lysosomal fluorescent membranes (i.e., a ring within a ring) following vacuolin-1 and LD staining. These structures are composed of portions of the plasma membrane, destined for degradation, that reach the lysosome in the form of small ILVs [88,89]. For DNR to reach and accumulate within these vesicles, it must first move through the luminal aqueous phase, hence being fully detached from the LLM. Apart from the LLM, ILVs present an additional accumulation domain for LDs that can contribute to the sequestration of, and consequently resistance to, LDs.

As mentioned above, although LDs cannot evade the lysosomal acidic trap by simple diffusion, they can be actively translocated from lysosomes into the cytoplasm by the Niemann-Pick C1 (NPC1) protein [90,91], a LLM transporter exporting cholesterol into intracellular destinations. The widely used lysosomal probes LTR and neutral red [92] were shown to undergo displacement from fibroblast lysosomes almost completely within 12 h, whereas there was little change in their lysosomal concentrations in NPC1 ^−/−^ cells within the tested 24 h period [90]. Except for the viable staining of lysosomes for their imaging, LTR has been used in several published assays as a tool to quantify the degree of lysosomotropism of a given compound [54,85,93,94]. These assays are based on the quantification of LTR displacement from lysosomes by exposure of the cells to increasing concentrations of the tested compounds. However, the exact mechanism of LTR displacement was not revealed. Benoit Lemieux et al., as well as Johannes Kornhuber et al., did explore the possible contribution of lysosomal alkalization by LDs to the lysosomal displacement of LTR but concluded that this is a pH-independent process [85,94]. The competitive nature of LTR displacement by other LDs indicates the involvement of a binding step in addition to the membrane insertion, as was previously suggested [32,86,87]. LTR reaches a steady-state equilibrium of ionic binding-dissociation to the phospholipid head group, which is disrupted at high competitor concentrations (i.e., >100 molar excess over LTR). With lower rebinding possibilities, it is readily available for clearance by NPC1. Taken together, these results suggest that hydrophobic weakly basic compounds can induce LTR displacement from lysosomes by enhancing NPC1 efflux activity. Indeed, several lysosomotropic amines were reported to stimulate the function of NPC1 in the clearance of lysosomal contents [90]. Enhanced membrane fluidization induced by highly membrane-accumulated LDs [22] might contribute to the increased activity of NPC1, as was shown for the G protein-coupled receptor serotonin 2B [95] and for epidermal growth factor receptor [96]. Another example of an LD as a substrate of NPC1 is the chemotherapeutic drug leelamine, a lipophilic diterpene amine phytochemical with a p*K*_a_ of 9.9, that was shown by molecular docking analysis to bind NPC1 at the cholesterol-binding pocket [97] and inhibit its activity in exporting cholesterol from lysosomes [98].

While LTR, LTG and DNR appear to exhibit an even lateral distribution within the round LLM, NTD induced distorted lysosomal membrane structures presenting uneven fluorescence distributions. Although the exposure time to NTD was quite short (i.e., 45 min), NTD accumulated in the lysosomal membrane at a high concentration to induce an impairment of the overall structure of the bilayer. It is possible that NTD accumulated in the LLM at concentrations that exceeded its solubility and hence precipitated. This hypothesis was proposed to account for the observation that certain hydrophobic amines accumulated in lysosomes at concentrations that were significantly higher than those computationally predicted [99,100]. If, indeed, NTD precipitated in the lysosomal membrane, it could account for the uneven NTD fluorescence captured by confocal microscopy imaging. MD simulations suggest that asymmetric drug incorporation into a lipid bilayer (i.e., confinement to only one leaflet), can induce local changes in membrane curvature [101]. All of the currently tested compounds are suggested to accumulate in the inner leaflet of the LLM; however, with the exception of NTD, they do not actually induce changes in lysosomal membrane morphology. Insertion analysis revealed that NTD, in its neutral form, was deeply inserted in the lipid tail region (around 12–15 Å from the membrane surface). Moreover, at the lowest pH studied (5.5) and contrarily to all other LDs, a significant amount of NTD was observed in its neutral state. The presence of both protonation forms of NTD at this low pH indicates that this LD can simultaneously inflict opposing effects at different membrane regions. On the one hand, NTD behaves like cholesterol that mostly accumulates at the tail regions of biological membranes, where it induces ordered domains that locally rigidify the bilayer structure [102,103], whereas at the water/membrane interface, it presumably induces membrane fluidization like SUN, SIR [22] and other LDs [104,105]. This dual effect is detrimental to the bilayer structure and to integral and peripheral proteins therein.

In addition to the membrane localization of LDs, predicted by various simulations, the accumulation of an assortment of amphiphilic weakly basic compounds in the inner leaflet of the LLM was suggested based upon their pharmacological effects. Specifically, the inhibition of intra-lysosomal enzymes, including acid sphingomyelinase (ASM) [56,57,106], acid ceramidase (AC) [107], and phospholipases A (PLA) and C (PLC) [108,109]. These lipases are transiently bound to the inner leaflet of the LLM via ionic interactions. The latter induce conformational changes in these enzymes and the consequent exposure of a hydrophobic surface, which allows the binding of the lipid substrate [110,111,112]. The ionic interactions were shown to depend on the presence of negatively charged phospholipids and an acidic pH, which were also required for the inhibitory effect of LDs on the above enzymatic activities [57,111,112,113]. The current view is based on the competitive binding of protonated LDs to the negatively charged phospholipids, which leads to neutralization of the charge of the inner LLM surface and consequent detachment of the above lysosomal lipases. Consequently, these lipases shift to their closed inactive forms, leading to a marked decrease in their enzymatic activities. Another contributing factor for the loss of these enzyme’s activities is the decrease in the protein levels of ASM, AC and PLA, presumably due to their subjection to intra-lysosomal proteolysis [107,112,114]. Impaired lipid catabolism leads to lysosomal lipid accumulation and consequent lipidosis, a hallmark of LSD [9]. A 2-day treatment with gentamicin, an antibiotic LD that causes renal impairment in ~30% of treated patients [115], induced renal cortical lysosomal phospholipidosis in adult rats, which was associated with decreased PLC activity [116]. This effect of LDs on membrane-bound enzymes is opposite to their impact on the lysosomal transmembrane protein NPC1 [117]. NPC1 is protected from the digestive environment of the lysosomal lumen by membrane phospholipids, whereas ASM, AC, PLA and membrane phospholipids are protected from proteolytic digestion by the lysosomal glycocalyx [88,118]. The glycocalyx is a thick coat of glycoproteins proposed to have an average thickness of ~8 nm, as determined by electron microscopy [119]. The carbohydrate luminal domains of lysosomal integral membrane proteins, together with various LLM-associated proteins, form a dense, highly *N*-glycosylated barrier, which is negatively charged and lines the inner surface of the LLM [118,120,121]. The apparent charge neutralization of the lysosomal luminal membrane leaflet surface by LDs disrupts major protein–lipid interactions and induces the collapse of the glycocalyx, following which ASM, AC and PLA become subjected to proteolysis by luminal enzymes.

Several amendments should be implemented in the above sequence of events. Negatively charged lipids—specifically, phosphtidylserine (PS), phosphatidylinositol (PI) and Bis(monoacylglycero)phosphate (BMP, lyso-bis-phosphatidic acid)—constitute only a small fraction of the total lysosomal lipidome (i.e., <10%) [122,123]. BMP is a unique negatively charged phospholipid found only in late endosomes and lysosomes [124]. It was found to be enriched 32-fold in the lysosome fraction over the homogenate of rat liver [125]; however, immuno-electron microscopy revealed that 99% of lysosomal BMP is localized to the ILV [126]. PS is mostly found in the inner leaflet of the plasma membrane [123], where it does not disturb the stability of the negatively charged glycocalyx on the outer cell surface [127]. It is reasonable to assume that PS does not reside in the inner leaflet of the LLM since it could disrupt the heavily negatively charged lysosomal glycocalyx. It is more likely that PS is a part of ILV membranes originating from the plasma membrane. Moreover, the incorporation of the negatively charged phospholipid phosphatidic acid into isolated lysosomes osmotically destabilized them [128], implying that the LLM should harbor only minute amounts of or no negative lipids at all. The requirement for negatively charged phospholipids for the membrane binding and activity of ASM, AC and PLA [57,111,112], together with the ILV localization of such lipids, implies that lipid catabolism occurs mainly or entirely on the surface of ILVs [88,89,129]. Since our confocal images show that LDs also accumulate in the membranes of ILV, it is easy to understand how LDs inhibit the activity of lysosomal lipases. The negative charge on the surface of ILVs is neutralized by the high concentration of positively charged LDs incorporated into the outer leaflet of the ILV bilayer, hence presumably disrupting the binding of lipases. Additionally, LDs could ionically bind negative lipids, hence masking lipid substrates from their specific lipases.

**Figure 7:**
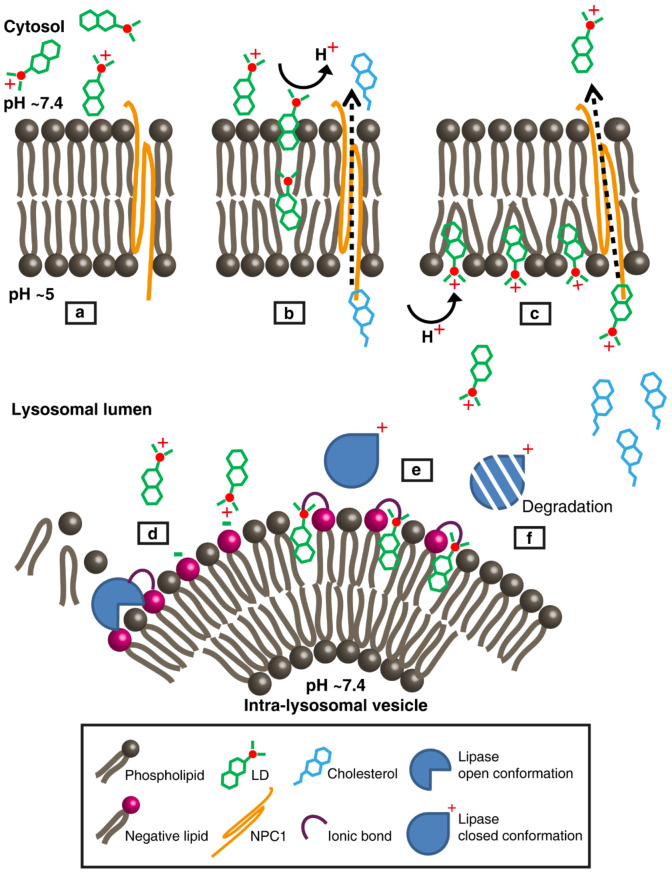
Model representation of lysosomotropic drug accumulation within the lysosomal membranes and its lysosomotropic effects. (**a**) A lysosomotropic drug (LD) reaches the lysosomal outer leaflet surface in its protonated form. The lysosomal limiting membrane (LLM) is illustrated with neutral phospholipids (e.g., phosphatidylcholine and sphingomyelin) and the transmembrane protein Niemann-Pick C1 (NPC1). For simplicity, the lysosomal glycocalyx is not shown. (**b**) The hydrophobic nature of the LD promotes its insertion into the bilayer, during which it undergoes complete deprotonation. Under physiological conditions, NPC1 exports cholesterol to the cytosol. (**c**) LD molecules diffuse through the LLM until their amine residues encounter the acidic lysosomal lumen, where they undergo protonation. As more LD molecules accumulate, they induce enhanced bilayer fluidity, thus enhancing NPC1 activity and competing with cholesterol for binding to NPC1. While most LD molecules concentrate within the inner leaflet of the LLM above the phospholipid head groups, some detach and move through the aqueous lumen. (**d**) The LD reaches the intra-lysosomal vesicle (ILV) surface, where various lipases (e.g., acid sphingomyelinase (ASM), acid ceramidase (AC) and phospholipase A (PLA)) are electrostatically bound to the membrane’s negatively charged lipids, while degrading their lipid substrates. (**e**) The LD inserts into the ILV’s outer leaflet and electrostatically interacts with negatively charged lipids, thereby abolishing the binding of lipases. (**f**) Lipases, in their closed-inactive form, are more rapidly degraded by luminal proteases. As a result of decreased lipase activity, phospholipids accumulate within the lumen of lysosomes, leading to lipidosis. The latter, along with the accumulation of cholesterol, is the hallmark of drug-induced and hereditary LSD.

Figure 7 depicts a proposed model summarizing the information gained by MD simulations, intra-lysosomal enzymatic pharmacology and the confocal imaging of LDs within lysosomes. Collectively, these combined results converge to the conclusion that LDs intercalate and accumulate in lysosomal membranes, hence inflicting their deleterious impact on the function of lysosomal enzymes.

In conclusion, lipophilic weak base therapeutics, known as LDs, undergo marked sequestration and concentration within lysosomal membranes, inducing alterations in membrane fluidity and integrity. The lysosomal membrane concentration of LDs provides the first molecular basis for the disruption of the lysosomal central signaling functions and cue sensing. Our findings bear important implications for lysosomal diseases and lysosomal damage as well as the future design of targeted therapeutic agents that evade lysosomal membrane entrapment

## Supplementary Materials

The following are available online at https://www.mdpi.com/2073-4409/9/5/1082/s1, Figure S1: Structure and physicochemical properties of various compounds employed in the current study, Figure S2: Increased size and number of LAMP1-mGFP labeled lysosomes following treatment with CNSDs, Figure S3: Representative quantification of the increase in lysosomal parameters following CNSDs treatment, Figure S4: ILVs contain marked levels of the anticancer drugs DNR and NTD, Figure S5: CpHMD simulations of membrane insertion of lysosomotropic anticancer drugs, Figure S6: Selected conformations of LDs inserted into a DMPC membrane model.
