# Supplementary material for: The Lysosomotropic Activity of Hydrophobic Weak Base Drugs is Mediated via Their Intercalation into the Lysosomal Membrane
Source: Cells. 2020 Apr 27;9(5):1082. doi: 10.3390/cells9051082 (PMC7290590; doi:10.3390/cells9051082)
Supplement: Supplementary file 1 [file cells-09-01082-s001.pdf]

Cells, 2020

The lysosomotropic activity of hydrophobic weak base drugs is mediated via their intercalation into the lysosomal membrane

Michal Stark, Tomás F. D. Silva, Guy Levin, Miguel Machuqueiro and Yehuda G. Assaraf

Supplementary Figures

Fig. S1: Structure and physicochemical properties of various compounds employed in the current study

Fig. S2: Increased size and number of LAMP1-mGFP labeled lysosomes following treatment with CNSDs

Fig. S3: Representative quantification of the increase in lysosomal parameters following CNSDs treatment

Fig. S4: ILVs contain marked levels of the anticancer drugs DNR and NTD

Fig. S5: CpHMD simulations of membrane insertion of lysosomotropic anticancer drugs

Fig. S6: Selected conformations of LDs inserted into a DMPC membrane model

Clomipramine
log P 5.19
pKa 9.5

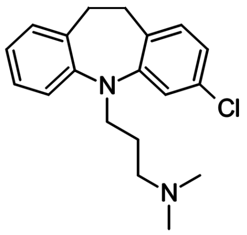

Pimozide
log P 6.3
pKa 8.63

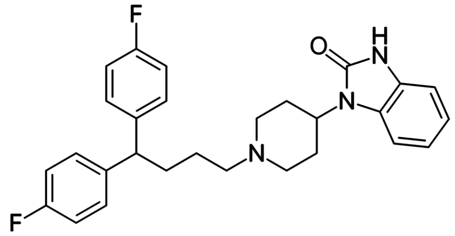

Ethopropazine
log P 4.77
pKa 9.64

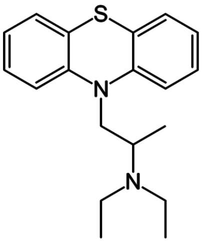

Chloroquine
log P 4.77
pKa 10.1

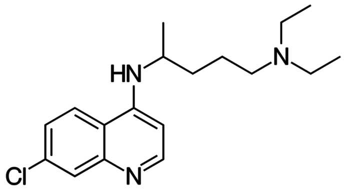

Sunitinib
log P 5.2
pKa 8.5

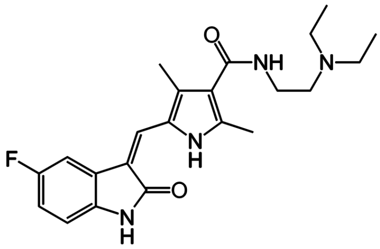

Nintedanib
log P 3
pKa 7.9

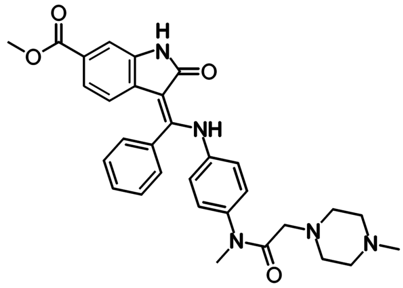

Daunorubicin
log P 1.83
pKa 8.46

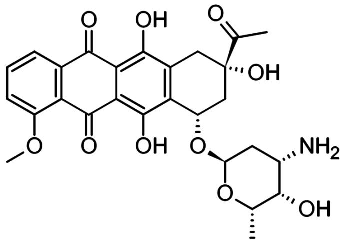

Vincristine
log P 2.82
pKa 8.31

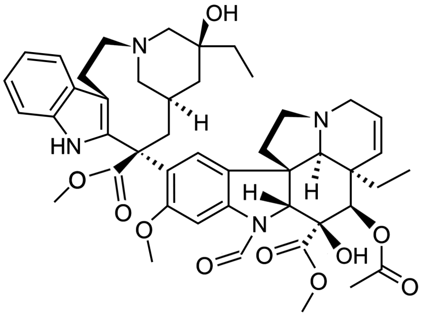

LysoTracker RED DND-99
log P 2.1
pKa 7.5

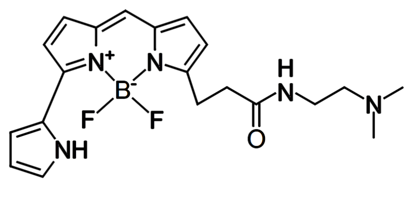

LysoTracker GREEN DND-26
log P U.P.
pKa U.P.

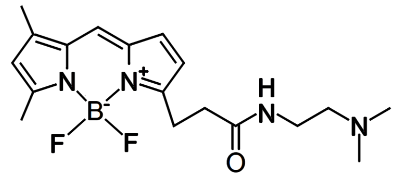

Fig. S1: Structure and physicochemical properties of various compounds employed in the current study. Experimental log P values were retrieved from the following publications; Pimo, Clomp, Ethop and CHQ [58]; SUN [53]; NTD [45]; DNR [59]; VCR [60]; and LTR [56]. Experimental pKa values were retrieved from the following publications; Clomp [49]; Pimo [50]; Ethop [51]; CHQ [52]; SUN [53]; NTD [45]; DNR [54]; VCR [55]; and LTR [56]. Values for LTG were never reported (unpublished U.P.).

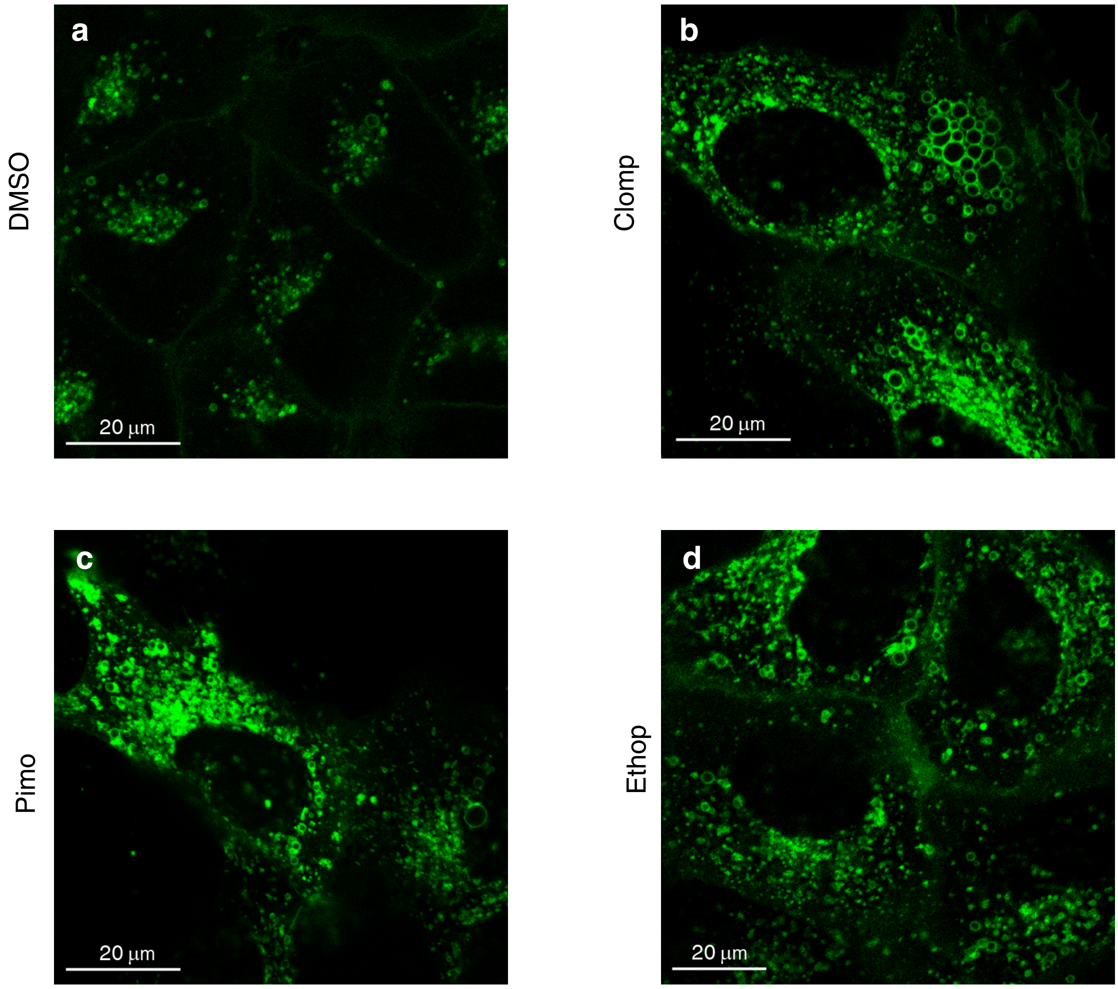

Fig. S2: Increased size and number of LAMP1-mGFP labeled lysosomes following treatment with CNSDs. U2OS cells stably expressing LAMP1-mGFP were treated for 72 h with either 10 μM Clomp, 10 μM Ethop, 30 μM Pimo or 0.1% DMSO as a drug-free control. Fluorescence was recorded using a confocal Zeiss LSM 710 microscope (×63 magnification).

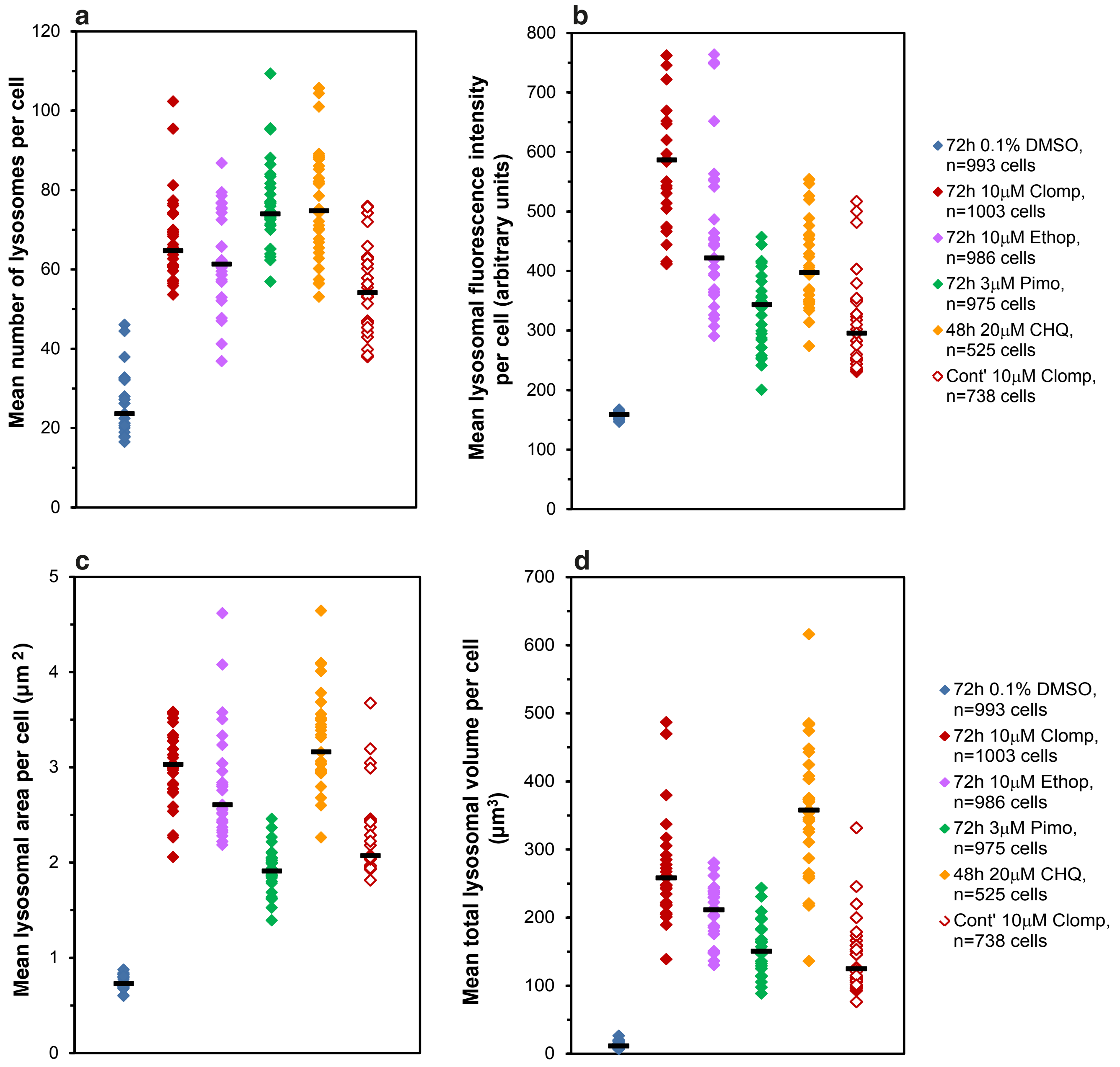

Fig. S3: Representative quantification of the increase in lysosomal parameters following CNSDs treatment. U2OS cells were seeded in black glass bottom plates and treated for 72 h with either 10 μM Clomp, 10 μM Ethop, 30 μM Pimo or 0.1% DMSO. In addition, ClompR cells continuously grown in 10 μM Clomp were used, as well as U2OS cells treated for 48 h with 20 μM CHQ as a positive lysosomotropic control. Nuclei were stained with the DNA dye Hoechst 33342 and lysosomes with the lysosomal probe LTR. Fluorescence was captured using an InCell Analyzer 2000 microscope, and lysosomes were analyzed using the InCell Investigator software. Each diamond represents the mean result of a microscope field, containing ~10-50 cells, where 32 independent fields were captured for each treatment from two 24-wells. The black bars denote the median results for each parameter. All p values within an experiment were < 5x10-12.

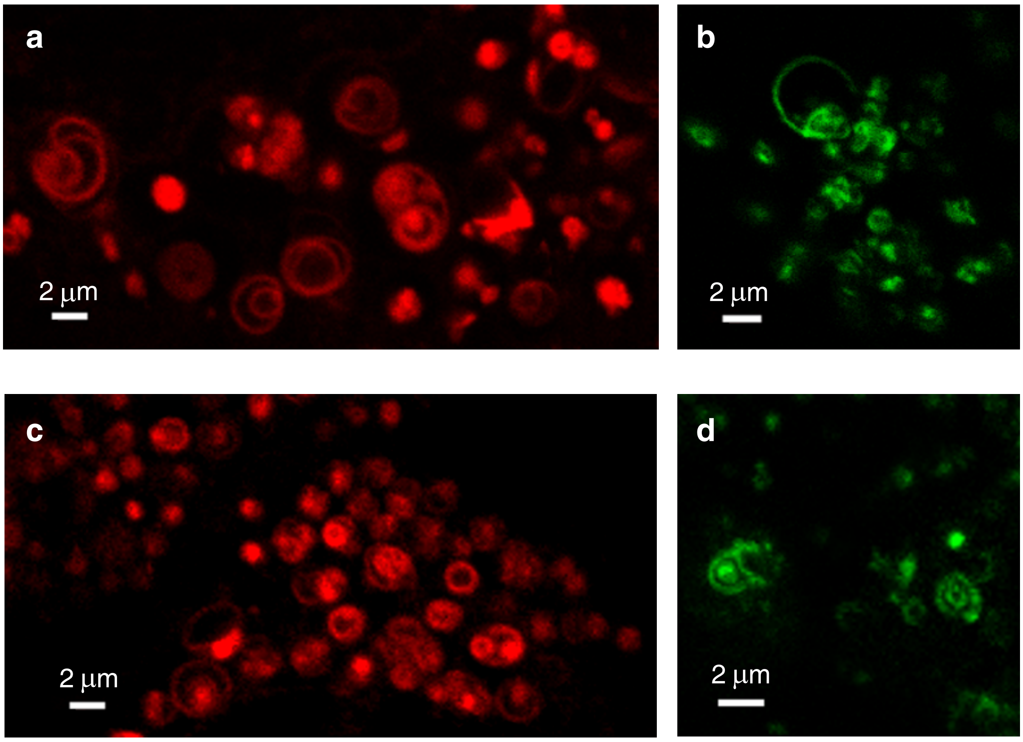

Fig. S4: ILV contain marked levels of the anticancer drugs DNR and NTD. Vacuolin-1-treated U2OS cells (a-b) and ClompR cells (c-d) were loaded for 45 min with DNR (a, c) or NTD (b, d) and captured with a confocal Zeiss LSM 710 microscope (×63 magnification).

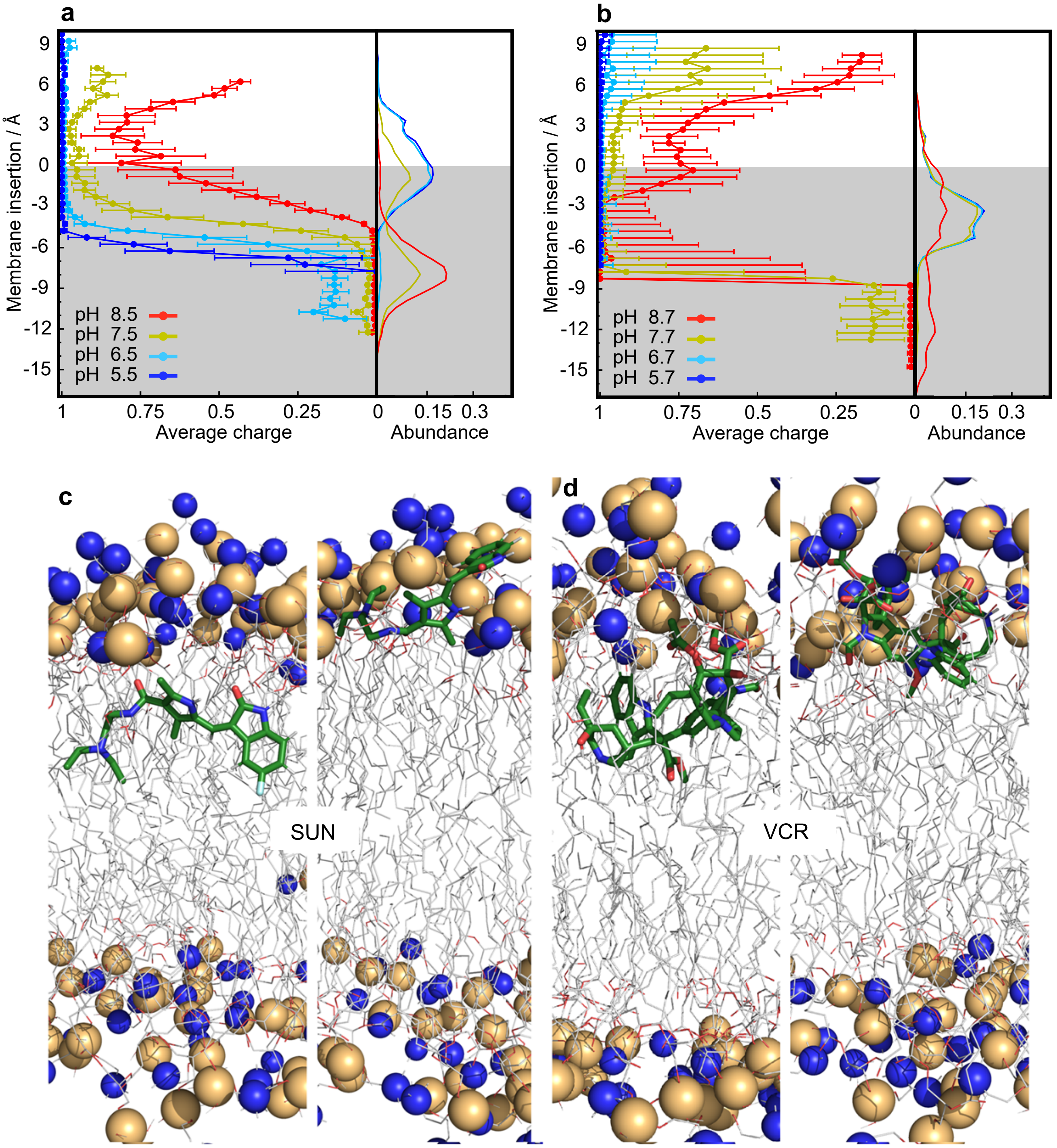

Fig. S5: CpHMD simulations of membrane insertion of lysosomotropic anticancer drugs. a-b, Average protonation (left subplots) and abundance histograms (right subplots) are depicted along the membrane insertion axis for Sun (a) and VCR (b). The grey-shaded area represents the membrane internal region below the phosphate groups, which are used as insertion references (see Materials and Methods). **c-d**, Selected conformations of SUN and VCR inserted into a DMPC membrane model are shown in two panels, with a more inserted location on the left panels and a shallower location on the right panels. The LDs are shown as sticks (green carbon atoms) and the phospholipids are shown in thin grey lines. The phosphorous and nitrogen atoms of DMPC are shown as orange and blue spheres, respectively, to highlight the polar region in the water/membrane interface.

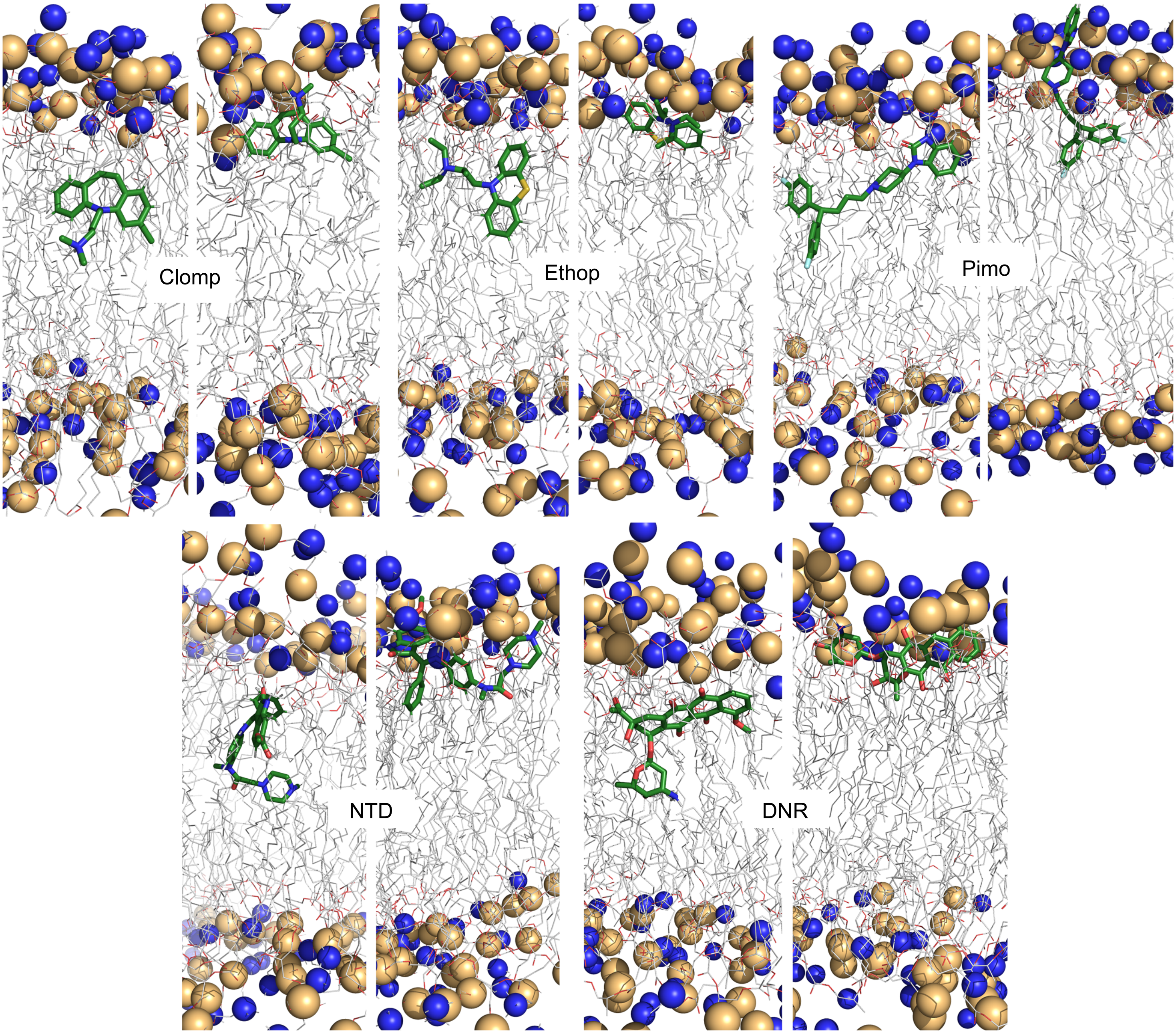

Fig. S6: Selected conformations of LDs inserted into a DMPC membrane model. Clomp, Ethop, Pimo, NTD and DNR are shown in two panels, with a more inserted location on the left panels and a shallower location on the right panels. The LDs are shown as sticks (green carbon atoms) and the phospholipids are shown in thin grey lines. The phosphorous and nitrogen atoms of DMPC are shown as orange and blue spheres, respectively, to highlight the polar region in the water/membrane interface.
